# Let's End HepC: Modelling Public Health Epidemiological Policies Applied to Hepatitis C in Spain

**DOI:** 10.3389/fpubh.2021.735572

**Published:** 2022-01-07

**Authors:** Henrique Lopes, Ricardo Baptista-Leite, Diogo Franco, Miguel A. Serra, Amparo Escudero, José M. Martín-Moreno

**Affiliations:** ^1^Institute of Health Sciences, Catholic University of Portugal, Lisbon, Portugal; ^2^Faculty of Health, Medicine and Life Sciences, Department of International Health, Care and Public Health Research Institute - CAPHRI, Maastricht University, Maastricht, Netherlands; ^3^Digestive Medicine Service, University of Valencia, Valencia, Spain; ^4^Department of Medicine, University of Valencia, Valencia, Spain; ^5^Department of Preventive Medicine and Public Health and INCLIVA, University of Valencia, Valencia, Spain

**Keywords:** hepatitis C, modelling, public health policies, public health, health literacy

## Abstract

**Background:** The WHO has defined international targets toward the elimination of hepatitis C by 2030. Most countries cannot be on track to achieve this goal unless many challenges are surpassed. The Let's End HepC (LEHC) tool aims to contribute to the control of hepatitis C. The innovation of this tool combines the modelling of public health policies (PHP) focused on hepatitis C with epidemiological modelling of the disease, obtaining a unique result that allows to forecast the impact of policy outcomes. The model was applied to several countries, including Spain.

**Methods:** To address the stated objective, we applied the “Adaptive Conjoint Analysis” for PHP decision-making and Markov Chains in the LEHC modelling tool. The tool also aims to be used as an element of health literacy for patient advocacy through gamification mechanisms and country comparability. The LEHC project has been conducted in several countries, including Spain. The population segments comprised in the project are: People Who Inject Drugs (PWID), prisoners, blood products, remnant population.

**Results:** A total of 24 PHP related to hepatitis C were included in the LEHC project. It was identified that Spain had fully implemented 14 of those policies to control hepatitis C. According to LEHC's model forecast, the WHO's Hepatitis C elimination goal on reducing the number of patients living with Hepatitis C to 10% can be achieved in Spain by 2026 if current policies are maintained. The model estimates that the total population in Spain, by 2026, is expected to comprise 26,367 individuals living with hepatitis C. Moreover, if the 24 PHP considered for this study are fully implemented in Spain, the elimination goal may be achieved in 2024, with 29,615 individuals living with hepatitis C by that year.

**Conclusion:** The findings corroborate the view that Spain has set great efforts in directing PHP toward Hepatitis C Virus (HCV) elimination by 2030. However, there is still room for improvement, namely in further implementing 10 of the 24 PHP considered for the LEHC project. By maintaining the 14 PHP in force, the LEHC model estimates the HCV elimination in the country by 2026, and by 2024 if further measures are employed to control the disease.

## Introduction

Worldwide, hepatitis C causes infections and profoundly harms the liver of thousands of people, being one of the leading causes of death related to hepatic complications (1). According to HCV's natural history, the disease can evolve toward varying degrees of liver damage and comorbidities over the years [e.g., fibrosis, cirrhosis, and hepatocellular carcinoma (HCC)] (2). This pathology results in high costs, such as liver transplants and other indirect expenditures (transmission pathways). The knowledge of hepatitis C epidemiological characteristics urges health services to allocate human resources, technology, and capital to control the disease.

A response to the coordination of PHP concerning the disease's epidemiological dynamics and contribution to the control of Hepatitis C is being promoted by the Public Health Unit of ICS/UCP, with the Let's End HepC (LEHC) project, properly aligning this with the sustainable development goals (SDGs) with HCV elimination targets being defined by the WHO (3).

The LEHC tool arises from the development from the classical works of Salomon (2, 4), in which it was possible to verify that it is possible to model the epidemiology of hepatitis C through the use of the natural history of the disease. This can be achieved by using Markov chains as an algorithmic tool, as the potential evolution of the disease throughout its natural history occurs in successive fractions (e.g., multipliers <0 resulting from the previous evolution state of the disease, ranging from the state of uninfected with transition to infected, to the state of death, which occurs in relatively low rate on the initial number of infected). This component of the LEHC does not add anything new to the literature, as it follows the protocol that all international teams follow while modelling hepatitis C. However, following the reading that micro-elimination would be a pivotal element to achieve the WHO's HCV elimination goals, the LEHC model was developed to comprise the introduction of this element as a new step in the modelling of hepatitis C, entailing the study of some population groups such as PWID, people who received blood products, vertical transmission, prisoners, among others (5).

The use of micro-segmentation in the LEHC model originates from raw epidemiological data. The second moment of profound innovation in the modelling of hepatitis is identified as incorporating public health policies (PHP) in the LEHC tool, expressed through 24 policies obtained in a literature review work and after discussion with 26 experts in hepatitis C. The integration of the impacts of PHP in the LEHC model, given their state of implementation in each country at a given date, acts on the raw results of the epidemiological model. This component allows to increase the model's fitting and, above all, allows to predict which PHP will impact hepatitis C more effectively and efficiently, in general, in each studied population group and at each year up to 2030, as any PHP is neither linear in its action nor does it impact equally on each subpopulation affected by the disease.

In sum, the research objective is to generate a forecast solution to HCV by combining epidemiological modelling and political decision-making focused on the disease [through Adaptive Conjoint Analysis (ACA)] while targeting micro-elimination in the general population and risk groups. Also, it is assumed in the field of health literacy that the involvement of patients, caregivers, and the community in general increases the notoriety involving the environmental context of any disease (12) and the patients' adherence to therapies, a situation that is particularly important in hepatitis C (13). One of the elements that limit this involvement and participation is the difficulty that many of the people who are patient advocates participate in Patients Associations, and patients themselves do not have the necessary technical knowledge to be able to properly defend their rights and duties, as well as how they can build realistic alternative solutions. The LEHC project also contributes to responding to the specific health literacy problem on hepatitis C, with different targets being identified for which it can be useful. Within these groups, we highlight health politicians and decision-makers who are not necessarily trained in Health and, as mentioned above, patient associations, patients, families, and caregivers.

The epidemiological traits of HCV prevalence values for chronic infections is between 0.5–3.5% in European countries (6, 7) and up to 7–8% in countries outside Europe (8). Revisions of these values set rates as low as 0.85–1.3% in population areas that have been systematically studied in Spain (9–11). Consequently, we have integrated Spain into the LEHC project's first phase country pool, along with other countries.

The choice for Spain to be included in a sample of 10 countries in the LEHC project resulted from being a country with high data consolidation complexity originating from the diversity of realities from its Autonomous Communities. This fact represented an insightful challenge for testing the LEHC model, as many other countries in the world also have some degree of decentralisation with consequent decision-making heterogeneity by regions within the Member State. These facts are reflected in PHP, including those specific for hepatitis C and the WHO's HCV elimination targets by 2030.

## Materials and Methods

### Context

The LEHC model was developed to obtain modelling scenarios by integrating, in a single solution, the impact of the study of PHP focused on HCV's epidemiological dynamics for every year until 2030 (14).

This article consisted of the practical application of the LEHC methodological article (14) to the Spanish context and other European countries, with each country being chosen due to a particular need for methodological development of its hepatitis C national context.

### Model Data Construction

In each country that participates in the LEHC project, a specific National Advisory Board (NAB) was constituted, composed of Key Opinion Leaders (KOLs) who are experts on HCV. Their contribution constitutes a pillar of the ACA system, one of the main components for modelling the impact of the 24 PHP (Table 5) that focused on HCV included in the LEHC model. Therefore, each policy has different application degrees, ranging from not existing to fully implemented, resulting in various impacts in specific Markov Chain links. As a result, it is possible to analyse the effect of all 24 PHP considered for this project concerning the epidemiological indicators for identified groups (PWID (15–17), prisoners (18–20), blood products (21, 22), vertical transmission, remnant population) and the sum of these population groups (total population).

We included a Spanish-based reading to decode the profound meanings of the country's regulatory framework (policies, available resources to control the disease, diversity of preventive and curative practises in Autonomous Regions, among other aspects) and HCV affected populations. The latter was crucial for analysing the existing epidemiological knowledge in the autonomous communities of the country. Moreover, we contextualised this information considering the objective and idiosyncratic behaviours of the populations and the observed behaviour of health organisations in their relationship with the patients of these populations. For this purpose, a scholarship was created for a young researcher (masters or doctoral student) to search and propose data sources for discussion and assist the NAB in the modelling of Spain data.

Each source was initially discussed with the Local Scientific Partner (INCLIVA—University of Valencia). If accepted, it was then proposed to the project's international scientific coordination. In this process, the data series' temporal extension was verified, along with the sources' reliability (official source, thesis, article, among others). When there were several alternatives for references, the choice was the one that simultaneously presented the best-perceived reliability, the comparison possibility with similar series in the project's country pool, and the ability to be worked on the model.

For each population segment worked on the model, a search for statistical series was conducted regarding Spain's general demographic data (e.g., age structure, gender, number of inhabitants, mortality, live births, among others), along with prevalence/incidence rates of hepatitis C, treatments, SVR, and mortality.

A total of around 200 statistical series were studied for its completeness, construction criteria by the issuing entity, data maintenance/transformation over the years, used metric units, and detected issues.

As in the remaining countries, it was necessary to generate proxies that comprised a statistical continuity of 65 years (1950–2015), in most series, to guarantee a sufficient number of freedom degrees for the model to work correctly. The resulting data was then discussed with the Spanish scientific partner. After achieving an agreement, we proposed the corresponding data for review/correction/replacement to the NAB. The main groups of statistical series, sources, proxies, Markov chain flowchart, PHP considered for the model, among other elements, can be consulted from the Transparency Appendix (Tables 1-5; Figures 2, 3).

Receiving the Scientific Partner and the NAB validation, modelling was conducted using Markov Chains, as initially theoretically proposed by Salomon et al. (4). This approach encompassed the population groups mentioned above, including the transition probabilities between health states, aligned with the HCV Cure Cascade and respecting the natural history of HCV presented by Salomon et al. (2), resulting in 1,100 active branches. We evaluated the results in the general population and each specific population according to the obtained fit with collected articles and other sources. We tried to make the model fit as closely as possible to the weighted average of available sources. As for the forecasting results, a continuity projection was made once the fit was established with the previous years' line.

The evaluation component of PHP focused on hepatitis C was conducted using ACA. This methodology is recognised (23–26) as the most efficient in capturing complex health choices. According to each respondent's answer patterns, the Sawtooth software generates a unique and specific ordering of questions from the 24 PHP considered for the project and their different scale degrees (calculated once we built the model). This response structure defines each policy's weight on the field according to each respondent's interpretation from a pool of 15 invited experts from Spain. Each electronic survey could have a maximum duration of 2 h when all the questions were responded to and the answers' complexity was high. The ordering of each battery's questions corresponded to the elements that the respondent knew best (years of work with each population group, typification of the respondent's role in the fight against hepatitis C (e.g., doctor vs. Patients' Association), among others).

The model arbitrarily attributes that the maximum impact of a means is 30% on the reality without PHP application. Therefore, each measure's impact varies in the algorithm between 0–30%, and experts then weighted according to the degree of importance attributed to each specific policy. This dual weighting classification system results in a statistical series' final impact between 0–30%. For this reason, the same policy approach can have a maximum effect on the general population and 0% in a population such as prisoners.

It is assumed that all experts dominate the hepatitis C subject regarding PHP focused on the disease. The scholarship holder was available to support the experts throughout the survey in clarifying issues, themes, among other aspects. For more complex questions, a help desk with doctorates was available to provide support to experts in a period within 24 h. Few questions have been asked on this platform.

### LEHC Model Features

Through the LEHC model's developed algorithm, we integrated the statistical series results with PHP decision-making. Finally, 14 indicators (total population; HCV incidence; HCV prevalence; diagnosed; linked to care; on treatment; cured; liver disease stages (from compensated cirrhosis to HCC); liver transplant; liver-related deaths; HCV elimination year) were calculated on the five population segments, and future needs to support the disease are estimated.

The definition of the HCV elimination target for the LEHC model was based on the set of targets defined by the WHO toward the elimination of HCV since 2015 (27), including the reduction of liver-related mortality by 65%, diagnosing 90% of individuals living with HCV infection, reduction of hepatitis C prevalence by 90%, among others. A 10% hepatitis C prevalence was defined as the elimination line for countries in the LEHC project due to being objective data of easier control. This criterion is widely applied in the modelling of the disease since there are many subjectivities in the classifications of liver-related deaths and difficulties in calculating diagnostic percentages. Still, the remaining elimination targets are also answered in the LEHC model through the output indicators. Since its genesis, the modelling of hepatitis C corresponds to its natural history and not to intermediate elements. Therefore, this article's methodology is aligned with the literature and the mathematical concept subject to modelling the disease.

To be understood by those interested in the hepatitis C subject who are unaware of the associated health contexts, it was considered crucial to attribute a technological opacity feature to the LEHC tool. Therefore, the LEHC aims to be a tool in which anyone can rehearse through a gamification system an arrangement of a set of 24 PHP considered for the LEHC project, the impact of these policies in hepatitis C for their country, being able to compare with other countries. Through this process, it is possible to verify the specific impact of a given policy on a given population to understand that, for example, a policy focusing on prisoners may have a relatively low impact on the country as a whole, but it may have a strong impact on the specific targeted population. The same is true for PWID or another population being tested. Also, the model's output systems provide objective indicators, as described above, of the consequences that the prevention of disease progression promoted by the cure for hepatitis C represents. For this reason, we consider that the LEHC tool allows for the health democratisation of technical discussions in the wider community, which discusses and includes those who work, suffer, and have to deal with hepatitis C. Therefore, there is a peer-to-peer logic between health professionals and patient associations/patients.

## Results

### Epidemiological Indicators

The model results for Spain appointed a total population of 46,796,432 individuals in 2019, with 172,048 being infected with HCV, mainly capturing active infection (HCV-RNA positivity), and an occurrence of 2,252 new cases to be registered. Forecasts show that 128,977 individuals were diagnosed that year, with 80,644 being linked to care, from which 36,332 were under treatment, and 29,458 cured cases were expected to happen. For the different liver disease stages, our model estimates 77,824 cases of compensated cirrhosis, 6,111 decompensated cirrhosis, and 3,275 cases of hepatocellular carcinoma (HCC). Also, 227 liver transplant cases and 3,745 liver-related deaths are expected.

In Spain, the LEHC model forecasts that HCV elimination will be achieved by 2026. According to the model, the total population is expected to comprise 46,225,796 individuals in 2026, from which 26,367 will be infected with HCV, and 1,945 are estimated to be new cases. Considering HCV infected individuals' pool, forecasts show that 21,909 are expected to be diagnosed, with 19,939 being linked to care. As a result, in 2026, 5,893 individuals are expected to be on treatment, with 7,147 individuals appointed to achieve the cured status. Regarding the liver disease stages, the model forecasts 68,757 cases of compensated cirrhosis, 2,364 cases of decompensated cirrhosis, and 1,505 HCC cases. Due to the burden of the disease, we expect that the health system will conduct 72 liver transplants, and it is estimated that there will be 1,735 cases of liver-related deaths. From 2026 until 2030, the model forecasts that further improvements are still verified on most epidemiological indicators.

### Modelled HCV Prevalence by Population

**Figure 1:**
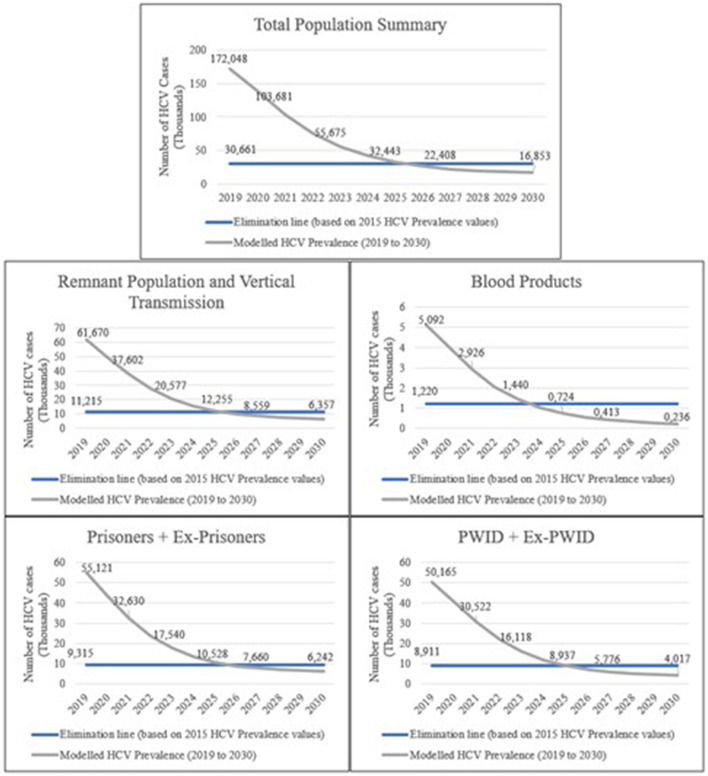
HCV prevalence model results for the studied populations in Spain. The modelled HCV prevalence values (thousands) represent the forecast for Spain if the current PHP focused on HCV remains unchanged until 2030. The results present a comparison with the elimination target for each studied population (total population, remnant population, vertical transmission, blood products, prisoners, PWID).

HCV prevalence model results for Spain indicate that the number of infection cases is expected to decrease steeply by 2030. The interpretation of this indicator in the LEHC model results appoints for the WHO target in Spain being expected to be achieved by 2026 (Figure 1).

Modelled HCV prevalence values for the remnant population (population of a country, excluding the remaining studied population groups in the model) and vertical transmission are expected to decrease until 2026 and slightly decline over the years steeply. As for the blood products population (individuals who received blood products before and after the implementation of mandatory HCV screening in blood donations), HCV prevalence is also expected to decrease over the years, with a steep decrease by 2024. HCV prevalence for the prisoners population is expected to steeply decrease until 2026 and slightly decrease over the remaining years. We also expect that HCV prevalence values for the PWID population will steeply decrease until 2025 and slightly decrease over the remaining years.

HCV incidence values between 2018 and 2030 are strongly influenced by specific incidences of prisoners and PWID, respectively. The accuracy of these values was considered in prison health reports and those related to PWIDs and Ex-PWIDs. However, we alerted that in several countries, the most recent epidemiological studies on prison populations show that the prevalence of hepatitis C is between 1/2 and 1/5 of what was expected (28), which we have also seen in global incidences. Likewise, the health management policy within prisons regarding hepatitis C will change the country's incidence, as prisoners and PWID represent very significant subpopulations of hepatitis C patients.

### Policies

Spain may further implement 10 from the 24 PHP considered in this study (Table 5.), as follows: National strategy that includes HCV (clinical evaluation and available resources evaluation); events or awareness campaigns for HCV; national register of disease for HCV; legal framework, particularly in terms of discrimination of HCV patients; national policy to address prevention of HCV infection; “Safe health” measures for screening and treatment of surgical instruments, equipment and supplies; general population screening; free and anonymous HVC testing, targeting high-risk populations; availability of social support for HCV patients. Nonetheless, if no changes are made to the current state of these measures, Spain is forecast to achieve the elimination of HCV by 2026.

By fully implementing all the 24 policies in Spain, the model results present the possibility of achieving HCV elimination by 2024. Model data indicates that a total population of 46,437,001 individuals is expected for 2024. HCV incidence values are expected to be 1,444 cases, and HCV prevalence values comprising 29,615 HCV infected individuals. The model also forecasts 26,217 diagnosed individuals, with 24,371 linked to care and 8,260 enrolled in treatment. In the same year, estimates indicate 11,149 cured cases. Concerning liver disease stages, the model predicts 71,605 compensated cirrhosis cases, 3,029 decompensated cirrhosis, and 1,774 individuals with HCC. We also estimate that 94 liver transplants will be conducted, and an occurrence of 2,158 liver-related deaths will be registered. Furthermore, the beneficial impact of implementing the 24 policies in Spain is also expected for subsequent years until 2030.

## Discussion

The modelling of hepatitis C has been worked with its basis on the natural history, an approach followed by Salomon et al. (2, 4). The very nature of subsequent work in this field has led to the development of other models with different designs and objectives (27, 29). The LEHC project has reinforced the evidence regarding the shift from medicines to achieve the HCV elimination by using an integrated solution emphasising the epidemiological modelling of the disease conjointly with measuring PHP significance. As this is a unique solution, the LEHC model outputs for Spain do not have a concurrent model to validate the obtained results.

Modelling different implementation degrees of 24 PHP in other population groups allows forecasting how a policy might impact HCV's epidemiological dynamics in a micro-elimination perspective. Also, the LEHC tool comprises a solution with total technological opacity, which is accessible by anyone who knows how to use a mobile phone or computer, being able to rehearse policies and verify the modelled results for hepatitis C.

It should be noted that Spain's greatly decentralised nature means that, although there are national policies, there are different applications for the same measure in the country's different regions. This work aims not to discuss how different applications of the same HCV targeted efforts in Spain's autonomous communities, as LEHC is an international project, and no country has been explored to this level of detail.

Nonetheless, efforts being allocated by Health Ministries and autonomous regions in Spain to achieve the HCV elimination goal are incredibly vigorous, with 14 fully implemented PHP, from a total of 24 measures being evaluated in this project. As a result, modelling for Spain forecasts the HCV elimination year as 2026, with the prospect of elimination set for 2024, only if the 24 PHP are fully implemented.

It is important to note that with the COVID-19 pandemic, efforts toward achieving the HCV elimination goal, such as in micro-elimination programmes (e.g., in PWID populations), are being affected due to multiple reasons: resource allocation for COVID-19 has led to a reduction of resources for other diseases, with significant importance for hepatitis C; the care for HCV infected patients often includes intubation procedures (e.g., endoscopy, colonoscopy) that generate aerosols, hence representing an increased risk for COVID-19 transmission among health professionals; the procedures reduction that will need to be compensated in the future will mean an aggravation of resource allocation (e.g., human, economic, and technical) for gastroenterology; some of COVID-19 infected patients may have related liver damage, which requires additional vigilance (30, 31).

Despite the importance of the COVID-19 dimension to hepatitis C, it was not included in the LEHC project, as the pandemic began even before the beginning of this work. However, we are aware that it is currently challenging to forecast the impact of COVID-19 in health systems and specific clinical areas, such as gastroenterology. In any case, we consider it imperative to alert about the threat that COVID-19 represents in this process and in maintaining all the efforts set in HCV elimination policies, especially in vulnerable populations, hoping that the pandemic will not be a very disruptive factor in achieving the WHO elimination goal for HCV.

### Research Limitations

The LEHC model is based on the assumption that policies are static for the future. To overcome this issue, through the estimation made from the experts' response structure, a simulation module was created where it is possible to test changes to policies and corresponding results.

It must also be considered that the modelling quality (either in LEHC or any other model) always depends on input data's availability and quality. The statistical data used for the model are assumed to be correct, to the extent that these are either published in peer-reviewed journals or official data from countries/regions. When none of these are available and proxies are required, we assumed that the country's leading experts involved in the LEHC project could provide an accurate interpretation of the national epidemiological reality. Nonetheless, it is vital to mention the difficulty of obtaining national data, which urged for the need to produce proxies based on Spanish regions data, extrapolate/interpolate national data, and create statistical series from proxies using other secondary data series.

The fact that the LEHC tool is essentially based on ICT ends up working only with info-included populations.

A call for research and publication of epidemiological data on HCV in Spain is necessary to fill in the data gaps. This project's scientific coordination had made it known to be fully available to integrate new data that the scientific community can make available to this modelling.

## Data Availability Statement

Modelling data outputs can be consulted on the project website while it is available (https://www.letsendhepc.com), after which the team is available to provide new modelling data. As for imputation data, the vast majority are available for public consultation as identified in the bibliography, while the remaining, namely those produced from proxies validated by the NAB, are available upon request.

## Author Contributions

HL: conceptualization, supervision, and project administration. HL and RB-L: methodology. DF: software. HL and DF: validation, data curation, and writing—original draft preparation. HL, RB-L, DF, MS, AE, and JM-M: investigation. MS, AE, and JM-M: writing—review and editing. All authors have read and agreed to the published version of the manuscript.

## Conflict of Interest

This study received funding from Gilead Sciences Europe Ltd. The funder had the following involvement with the study: financial support for the national data collection part of the modelling, web design, and IT of the LEHC project. Article authorship, national advisory board, and scientific/academic work are not sponsored. All authors declare no other competing interests.

## Publisher's Note

All claims expressed in this article are solely those of the authors and do not necessarily represent those of their affiliated organizations, or those of the publisher, the editors and the reviewers. Any product that may be evaluated in this article, or claim that may be made by its manufacturer, is not guaranteed or endorsed by the publisher.
