# Supplementary material for: Let's End HepC: Modelling Public Health Epidemiological Policies Applied to Hepatitis C in Spain
Source: Front Public Health. 2022 Jan 7;9:735572. doi: 10.3389/fpubh.2021.735572 (PMC8777247; doi:10.3389/fpubh.2021.735572)
Supplement: Supplementary file 1 [file Data_Sheet_1.docx]

Let’s End HepC: Modelling public health epidemiological policies applied to hepatitis C in Spain

Henrique Lopes^1^, Ricardo Baptista-Leite^1,2*^, Diogo Franco^1^, Miguel A. Serra^3^, Amparo Escudero^4^, José M. Martín-Moreno^5^

^1^Institute of Health Sciences, Universidade Católica Portuguesa (Catholic University of Portugal). Lisbon. Portugal.

^2^Faculty of Health, Medicine and Life Sciences, Maastricht University. Maastricht. The Netherlands.

^3^Digestive Medicine Service. University of Valencia. Valencia. Spain.

^4^Medicine Department. University of Valencia. Valencia. Spain.

^5^Department of Preventive Medicine and Public Health & INCLIVA, University of Valencia. Valencia. Spain.

*** Correspondence:**Henrique Lopes
henrique.lopes@ucp.pt

Keywords: Hepatitis C, Modelling, Public Health Policies, Public Health, Health Literacy

# Supplementary Material

## Appendix A - Statement of Transparency on origin and use of data

**Table 1.** Data support and construction of Remnant Population.

| **Remnant Population** | | | |
| --- | --- | --- | --- |
| **Statistical Series** | **Source** | **Features** | **Proxies** |
| Demography | - Instituto Nacional de Estadística. Resident population by date, sex and age [Internet]. Main series since 1971 - National results. 2017 [cited 2019 Sep 4]. Available from: <http://www.ine.es/jaxiT3/Tabla.htm?t=10256>  - Landwerlin GM. La población española. Acento, editor. 1999. | Data obtained regarding the evolution of live births and population structure by age groups and gender. | Between 1950 to 1971, a proxy was produced to align values with the following years found in data. |
| Background Mortality | - Instituto Nacional de Estadística. Vital Statistics: Deaths (Annual figures) [Internet]. By place of residence, sex and age. National total and provinces. 2017 [cited 2019 Sep 4]. Available from: https://www.ine.es/jaxiT3/Tabla.htm?t=6547&L=1 | Data obtained for the period between 1975 and 2017. | For the period between 1950 and 1974, the values were assumed to be the same as in the year 1975, while for the period between 2018 and 2030, the values were considered the same as in the year 2017. |
| HCV Incidence | - Rituerto AC, Taltavull TC. Hepatitis por el virus de la Hepatitis C. Control Calid SEIMC. 2000;1–11.  - Bruguera M, Forns X. Hepatitis C en España. Med Clin (Barc) [Internet]. 2006;127(3):113–7. Available from: http://dx.doi.org/10.1157/13090276  - iberoaméricadivulga. Dr. Xavier Forns: “Un paciente curado de Hepatitis C está curado al 100%” [Internet]. 2013. Available from: https://www.oei.es/historico/divulgacioncientifica/?Dr-Xavier-Forns-Un-paciente-curado | HCV Incidence rates have gone from 6,8 per 100.000 inhabitants in 1997 to 2,3 in 2003, with 2.600 new HCV infections identified in 2015. | According to clinical data, values were calibrated and aligned with the Local Research Team’s indication in Spain, indicating there would be 1.000 new HCV cases in 2018. A proxy was generated to progressively decrease between 1950 and 2018, aligned with the aforementioned incidence rates. |
| HCV Prevalence | - Rituerto AC, Taltavull TC. Hepatitis por el virus de la Hepatitis C. Control Calid SEIMC. 2000;1–11.  - iberoaméricadivulga. Dr. Xavier Forns: “Un paciente curado de Hepatitis C está curado al 100%” [Internet]. 2013. Available from: https://www.oei.es/historico/divulgacioncientifica/?Dr-Xavier-Forns-Un-paciente-curado | - | A proxy was produced for the period between 1950 and 2018, considering estimated values that correspond to the HCV Prevalence average provided by the Local Research Team in Spain, mentioning a total of 174.000 HCV infected individuals to date. |
| Vertical Transmission | Ruiz-Extremera Á, Díaz-Alcázar MdM, Muñoz-Gámez JA, Cabrera-Lafuente M, Martín E,  Arias-Llorente RP, et al. (2020) Seroprevalence and epidemiology of hepatitis B and C viruses in pregnant women in Spain. Risk factors for vertical transmission. PLoS ONE 15(5): e0233528. https://doi.org/10.1371/journal.pone.0233528  - Linares, A., Serrano, N., Higuera, G., Pinedo, B. (2016) Hepatitis C en Pediatría. Form Act Pediatr Aten Prim. 9(2):71-5 (http://archivos.fapap.es/files/639-1370-RUTA/03_Hepatitis_C.PDF)  - Ruiz-Extremera Á, Salmerón J. Transmisión del virus de la hepatitis C al recién nacido. GH Contin. 2003;2(5):225–9.  - De A, Bueno V, Remacha F. Conducta ante la hepatitis por el VHC El virus. 2000. p. 203–12. | - | Values for this indicator represented an annual variation between 360 and 700 HCV infection cases until 2014, with the following years reflecting a significant reduction in Vertical Transmission cases to around 40 HCV-infected live births. |
| Diagnose and RIC | - Ministerio de Sanidad, Servicios Sociales e Igualdad. Informe de situación del plan estratégico para el abordaje de la hepatitis c crónica presentado al consejo interterritorial del SNS. 2017.  - Arenas C. Current Situation in Hepatitis C Drug Treatment Difficulties in Spain. FarmaJournal. 2016;1(2):165–9. | - | It was assumed that HCV diagnosis and retain in care (RIC) measures were implemented since 1994, with values for previous years considered to be 0%. The accumulated value regarding Diagnose was defined at 50% over the HCV infection cases, with a higher significance in the post-DAA phase, given the considerable efforts towards finding new patients. As for RIC, the accumulated value was defined at 48% over the HCV infection cases between 1994 and 2013. A higher significance was found in the post-DAA phase (90%), given the considerable efforts to maintain new patients in the health system. |
| Tx1 and Tx2 | - Mínguez C, García-Deltoro M, Flores J, Galindo M-J, Montero M, Reus S, et al. Interferon-free therapy for treating HCV in difficult-to-treat HIV-coinfected patients as implemented in routine medical practice. AIDS. 2018;1:1–34.  - Wyles D, Poordad F, Wang S, Alric L, Felizarta F, Paul Y, et al. Glecaprevir/Pibrentasvir for HCV Genotype 3 Patients with Cirrhosis and/or Prior Treatment Experience: A Partially Randomised Phase III Clinical Trial. Hepatology. 2018;67(2):514–23.  - González-Grande, R., Jiménez-Pérez, M., González Arjona, C., & Mostazo Torres, J. (2016). New approaches in the treatment of hepatitis C. World Journal of Gastroenterology, 22(4), 1421–1432. <https://doi.org/10.3748/wjg.v22.i4.1421>  - Poordad, F., Pol, S., Asatryan, A., Buti, M., Shaw, D., Hézode, C., … Mensa, F. J. (2018). Glecaprevir/Pibrentasvir in patients with hepatitis C virus genotype 1 or 4 and past direct-acting antiviral treatment failure. Hepatology, 67(4), 1253–1260. https://doi.org/10.1002/hep.29671 | - | The probability of treating new patients if retained in care reaches a rate of 50% for the period between 1996 and 2014. For the period between 2015 and 2018, the probability of treatment was considered to increase by up to 90%. The annual probability of retreating a patient if retained in care is applied since 2015, considering a rate of 93% for this period. |
| SVR1 and SVR2 | - Butt AA, Yan P, Simon TG, Abou-Samra A-B. Effect of Paritaprevir / Ritonavir / Ombitasvir / Dasabuvir and Ledipasvir / Sofosbuvir Regimens on Survival Compared With Untreated Hepatitis C Virus – Infected Persons : Results From ERCHIVES. Clin Infect Dis. 2017;65(6):1006–11.  - Forns X, Lee SS, Valdes J, Lens S, Ghalib R, Aguilar H, et al. Glecaprevir plus pibrentasvir for chronic hepatitis C virus genotype 1 , 2 , 4 , 5 , or 6 infection in adults with compensated cirrhosis (EXPEDITION-1): a single-arm, open-label, multicentre phase 3 trial. Lancet Infect Dis [Internet]. 2017;17(10):1062–8. Available from: http://dx.doi.org/10.1016/S1473-3099(17)30496-6  - Zeuzem S, Ghalib R, Reddy KR, Pockros PJ, Ari Z Ben, Zhao Y, et al. Grazoprevir–Elbasvir Combination Therapy for Treatment-Naive Cirrhotic and Noncirrhotic Patients With Chronic HCV Genotype 1, 4, or 6 infection. Ann Intern Med. 2015;163(1):1–23.  - Asociación Española para el estudio del hígado. Documento del II Consenso español sobre tratamiento de la hepatitis C. 2015.  - Berenguer J, Gil-Martin Á, Jarrin I, Moreno A, Dominguez L, Montes M, et al. All-Oral Direct-Acting Antiviral Therapy Against Hepatitis C Virus (HCV) in Human Immunodeficiency Virus/HCV-Coinfected Subjects in Real-World Practice: Madrid Coinfection Registry Findings. Hepatology. 2018;68(1):32–47. | - | Since 1996 and until the implementation of DAA treatments, the SVR1 (Sustained Virologic Response) rate was considered to be 50%. Since 2014, an SVR of 92% was observed in new and pretreated patients, with or without overt cirrhosis.  As for the SVR rates for patients being retreated, it is expected that the cure in patients without cirrhosis is 100% by the year 2030. Rates remained above 90% in all participants without liver cirrhosis and 72,7% for patients with cirrhosis between 2016 and 2018. |

**Table 2.** Data support and construction of PWID Population.

| **PWID Population** | | | |
| --- | --- | --- | --- |
| **Statistical Series** | **Source** | **Features** | **Proxies** |
| Demography | - European Monitoring Centre for Drugs and Drug Addiction. España: Informe del país sobre drogas 2017. 2017. | - | For the period between 1961 and 2030, on the total PWID population distribution, it was assumed that the ratio between genders should comprise 80% for male individuals and 20% for females. This distribution was also applied in the PWID’s Turnover, HCV Incidence, and HCV Prevalence. |
| Turnover | - Roncero C, Littlewood R, Vega P, Martinez-raga J, Torrens M. Chronic hepatitis C and individuals with a history of injecting drugs in Spain: population assessment, challenges for successful treatment. Eur J Gastroenterol Hepatol. 2017;29(6):629–33.  - European Monitoring Centre for Drugs and Drug Addiction. España: Informe sobre drogas 2018. 2018. | - | The Turnover-in for this population was based on a distribution of HCV Incidence aligned with the prevalence value of 300.000 HCV infected individuals in 2013. For the Turnover-Out, it was considered that 75% to 90% of the PWID population was enlisted in OST programs. The ones that keep injecting drugs consume an average of 1.5 million syringes per year, which means that there are between 10.000 to 15.000 active PWIDs in syringe exchange programs. A peak of injectable drug incidence in Spain was considered, between the 1980s and the early 1990s, accounting for the return cycle into the social setting over the years. Therefore, the distribution of incidence values was aligned with the found prevalence rates. |
| Background Mortality | - Brugal MT, Barrio G, Royuela L, Bravo MJ, Fuente L de la, Regidor E. Estimación de la mortalidad atribuible al consumo de drogas ilegales en España. Med Clin (Barc). 2004;123(20):775–7.  - Lumbreras B, Jarrı I, Amo J del, Pérez-Hoyos S, Muga R, Hera MG la, et al. Impact of hepatitis C infection on long-term mortality of injecting drug users from 1990 to 2002: differences before and after HAART. AIDS. 2006;20(1):111–6.  - Mathers BM, Degenhardt L, Bucello C, Lemon J, Wiessing L, Hickman M. La mortalidad entre consumidores de drogas inyectables: una revisión sistemática y meta-análisis [Internet]. Boletín de la Organización Mundial de la Salud 91(2). 2013 [cited 2019 Sep 4]. Available from: https://www.who.int/bulletin/volumes/91/2/12-108282-ab/es/ | Mortality rates went from 22,7% in 1996 to 8,9% in 2009. | For the period between 1950 and 1974, the values were assumed to be the same as in the year 1975, while for the period between 2018 and 2030, the values were considered the same as in the year 2017. |
| HCV Incidence and Prevalence | - European Monitoring Centre for Drugs and Drug Addiction. España: Informe del país sobre drogas 2017. 2017.  - European Monitoring Centre for Drugs and Drug Addiction. España: Informe sobre drogas 2018. 2018.  - Saludes V, Folch C, Antuori A, González N, Ibáñez N, Colom J, et al. A one-step diagnosis algorith reveals high burden of hepatitis among PWID in Spain and the urgency for improved linkage-to-care. 2017 p. 1–4. | HCV prevalence rates were found to be 66,6%, with some references to reactive HCV antibodies and others that identified 65% of active HCV. | HCV Incidence values were calculated by considering that a rate of 67% should be applied to each year and all age groups in this population’s Turnover-in.  Considering the identified literature, a rate of 67% regarding HCV Prevalence was applied since 1950. |
| Diagnose and RIC | - Ministerio de Sanidad, Servicios Sociales e Igualdad. Informe de situación del plan estratégico para el abordaje de la hepatitis c crónica presentado al consejo interterritorial del SNS. 2017.  - Arenas C. Current Situation in Hepatitis C Drug Treatment Difficulties in Spain. FarmaJournal. 2016;1(2):165–9. | - | Since 1994 diagnosis rates were produced according to an accumulated value regarding 50% over the HCV infection cases of the Remnant Population. A higher significance was set in the post-DAA phase, given the considerable efforts towards finding new patients. The same procedure was applied to RIC, with an accumulated value of 48% being considered for 1994 and 2013. For previous years rates for this indicator were assumed as being 0%. |
| Tx1, Tx2, SVR1, SVR2 | - Mínguez C, García-Deltoro M, Flores J, Galindo M-J, Montero M, Reus S, et al. Interferon-free therapy for treating HCV in difficult-to-treat HIV-coinfected patients as implemented in routine medical practice. AIDS. 2018;1:1–34.  - Wyles D, Poordad F, Wang S, Alric L, Felizarta F, Paul Y, et al. Glecaprevir/Pibrentasvir for HCV Genotype 3 Patients with Cirrhosis and/or Prior Treatment Experience: A Partially Randomised Phase III Clinical Trial. Hepatology. 2018;67(2):514–23.  - González-Grande, R., Jiménez-Pérez, M., González Arjona, C., & Mostazo Torres, J. (2016). New approaches in the treatment of hepatitis C. World Journal of Gastroenterology, 22(4), 1421–1432. <https://doi.org/10.3748/wjg.v22.i4.1421>  - Poordad, F., Pol, S., Asatryan, A., Buti, M., Shaw, D., Hézode, C., … Mensa, F. J. (2018). Glecaprevir/Pibrentasvir in patients with hepatitis C virus genotype 1 or 4 and past direct-acting antiviral treatment failure. Hepatology, 67(4), 1253–1260. https://doi.org/10.1002/hep.29671 | - | Values for this indicator were considered to be equal to the same statistical series in Remnant’s population. |

**Table 3.** Data support and construction of Prisoners’ Population.

| **Prisoner’s Population** | | | |
| --- | --- | --- | --- |
| **Statistical Series** | **Source** | **Features** | **Proxies** |
| Demography and Background Mortality | - Cid J. El incremento de la población reclusa en España entre 1996-2006: Diagnóstico y remedios. Rev Española Investig Criminológica. 2008;6(2):1–31.  - García-guerrero J, Vera-Remartínez EJ, Planelles M V. Causes and Trends of mortality in a Spanish Prison ( 1994-2009 ). Rev Esp Salud Publica. 2011;85(3):245–55.  - Remartínez EJV, Ramos MVP, Guerrero JG. Trends in Mortality in a Spanish Prison from 1994-2004. Rev Esp Salud Publica. 2005;79(6):673–82. | For the period between 1996 and 2006, a study presents the total prisoner population in Spain for those years.  The mortality rate evolution was based on values for the period between 1994 and 2009. | In 1975 and 1978, pardons were decreed to prisoners. For example, the population in 1975 is lower than in previous years due to the General Decree of pardon 2940/1975, 25th November. In 1982 a limit was placed on the time that a person could be a pre-trial prisoner (two years). Many prisoners who were awaiting trial and had been in prison for more than two years had to be released. Since 1984, Cataluña has full powers in prison matters, treating and analysing data in different ways. In 2012 a methodological modification of the Statistics of the Prisoner Population data source came into effect within the General Secretariat for Prison Affairs scope. The Prison Information System replaces the statistical questionnaires completed by the Centers Penitentiaries. In 2015 a Criminal Code reform came into force, eliminating offences and turning them into minor offences.  For the years following 2018, a decrease rate was estimated by comparing the previous five years, being applied an annual decrease of 3,52%.  A proxy was generated to maintain the evolution of the mortality rate in previous and subsequent years. |
| HCV Incidence | - García-guerrero J, Vera-Remartínez EJ, Planelles M V. Causes and Trends of mortality in a Spanish Prison ( 1994-2009 ). Rev Esp Salud Publica. 2011;85(3):245–55.  - Remartínez EJV, Ramos MVP, Guerrero JG. Trends in Mortality in a Spanish Prison from 1994-2004. Rev Esp Salud Publica. 2005;79(6):673–82.  - Marco A, Guerrero R, Turu E, Gallego C, Teixidó N, Sastre A, et al. Is it possible to eliminate hepatitis C from the prisons of Catalonia, Spain, in 2021? Rev Española Sanid Penit. 2019;21(1):38–41.  - Mouriño AM. Incidencia de la hepatitis crónica por VHC y necesidad de tratamiento en los internados de prisión. Rev Española Sanid Penit. 2006;8(3):71–7.  - Denniston MM, Jiles RB, Drobeniuc J, Klevens RM, Ward JW, McQuillan GM, et al. Chronic Hepatitis C Virus Infection in the United States, National Health and Nutrition Examination Survey 2003 to 2010. Ann Intern Med. 2014;160(5):293–300. | - | HCV Incidence was based on the literature, with the Local Research Team in Spain indicating that it corresponded to eight times the average HCV Incidence of the General Population. |
| HCV Prevalence | - Cuadrado A, Llerena S, Cobo C, Pallás JR, Mateo M, Cabezas J, et al. Microenvironment Eradication of Hepatitis C: A Novel Treatment Paradigm. Am J Gastroenerology. 2018;1–10.  - Arroyo-cobo JM, Hernández-Fernández T. Results of the Spanish experience: A comprehensive approach to HIV and HCV in prisons. Rev Española Sanid Penit. 2010;12(3):86–90.  - Grupo Noroeste para el estudio de la Hepatitis por Virus C en el medio penitenciario. Seroprevalencia de infección por virus C de la Hepatitis en poblácion reclusa del noroeste de españa a su ingreso en prisión. Rev Esp Salud Publica. 1998;72(1):43–51. | HCV Prevalence values were aligned with a rate of 48%. Also, HCV Prevalence had been reduced from 38% in 2003 to 11,3% in 2016. For the period between May 2016 and July 2017, the HCV antibodies rate was found to be 13%, while 10,2% had detectable viremia. | A proxy was generated to apply a decrease rate from 1950 to 1991. A proxy was generated to align the values with a gradual decrease of the intervening years. |
| Diagnose and RIC | - Ministerio de Sanidad, Servicios Sociales e Igualdad. Informe de situación del plan estratégico para el abordaje de la hepatitis c crónica presentado al consejo interterritorial del SNS. 2017.  - Arenas C. Current Situation in Hepatitis C Drug Treatment Difficulties in Spain. FarmaJournal. 2016;1(2):165–9. | - | Diagnosis and RIC rates were assumed to be 0% before 1994. Since that year, diagnosis values were considered until achieving an accumulated rate of 95% over the Remnant population’s HCV infection cases. A higher significance was set in the post-DAA phase, given the considerable efforts towards finding new patients. The same procedure was applied to RIC, with an accumulated value of 48% being considered for 1994 and 2014, which raised for up to 90% in subsequent years. |
| Tx1, Tx2, SVR1, SVR2 | - Mínguez C, García-Deltoro M, Flores J, Galindo M-J, Montero M, Reus S, et al. Interferon-free therapy for treating HCV in difficult-to-treat HIV-coinfected patients as implemented in routine medical practice. AIDS. 2018;1:1–34.  - Wyles D, Poordad F, Wang S, Alric L, Felizarta F, Paul Y, et al. Glecaprevir/Pibrentasvir for HCV Genotype 3 Patients with Cirrhosis and/or Prior Treatment Experience: A Partially Randomised Phase III Clinical Trial. Hepatology. 2018;67(2):514–23.  - González-Grande, R., Jiménez-Pérez, M., González Arjona, C., & Mostazo Torres, J. (2016). New approaches in the treatment of hepatitis C. World Journal of Gastroenterology, 22(4), 1421–1432. <https://doi.org/10.3748/wjg.v22.i4.1421>  - Poordad, F., Pol, S., Asatryan, A., Buti, M., Shaw, D., Hézode, C., … Mensa, F. J. (2018). Glecaprevir/Pibrentasvir in patients with hepatitis C virus genotype 1 or 4 and past direct-acting antiviral treatment failure. Hepatology, 67(4), 1253–1260. https://doi.org/10.1002/hep.29671 | - | Values for this indicator were considered to be equal to the same statistical series in Remnant’s population. |

**Table 4.** Data support and construction of Blood Products Population.

| **Blood Products Population** | | | |
| --- | --- | --- | --- |
| **Statistical Series** | **Source** | **Features** | **Proxies** |
| Demography | - Peacock A, Gibbs D, Karlsson A, Sutherland R, Bruno R, Lenton S, et al. Australian drug trends 2018: Key Findings from the national ecstasy and related drugs reporting system (EDRS) Interviews. Sydney; 2018. | Statistical series for this population includes people who received blood transfusions before and after the implementation of obligatory HCV tests in blood donations. | This population’s demography is based on a proxy produced by considering the Spanish population by gender ratios: 56% correspond to male individuals while 44% to female individuals. |
| Turnover | - Ministerio de Sanidad SSEI. Plan estratégico para el abordaje de la hepatitis c en el sistema nacional de salud. 2015.  - Sociedad Española de Transfusión Sanguínea. ALT y las barbas del vecino. 2006;18(3):1–40.  - Sociedad Española de Transfusión Sanguínea. Su turno. 2007;19(4):1–24.  - Sociedad Española de Transfusión Sanguínea. Relevo en la presidencia de la SETS. 2005;57(3). | It was found that there is an average of 10% related to a new annual income of individuals into this population. It was also considered that the population who has entered the Blood Products population would never leave it. A percentage of 0% for the Turnover-out was applied in the period between 1950 and 2030. | Considering this value, a proxy was produced for the Turnover-in in the period between 1950 and 1990. For the following years, it was considered that after the implementation of HCV screening in blood products, the values for this indicator should be close to zero. Therefore, the population that receives any of these products is still considered for the model calculations, given that individuals still go through that clinical act. At the same time, an exclusion factor is applied in almost the total population in this period, considering that the infection risk by false negative would be 2/100.000. |
| Background Mortality | - Peacock A, Gibbs D, Karlsson A, Sutherland R, Bruno R, Lenton S, et al. Australian drug trends 2018: Key Findings from the national ecstasy and related drugs reporting system (EDRS) Interviews. Sydney; 2018. | Mortality rates went from 22,7% in 1996 to 8,9% in 2009. | This population’s background mortality values are based on a proxy produced by considering the Spanish population by gender ratios: 56% correspond to male individuals, while 44% to female individuals. |
| HCV Incidence and Prevalence | - Ministerio de Sanidad SSEI. Plan estratégico para el abordaje de la hepatitis c en el sistema nacional de salud. 2015.  - Sociedad Española de Transfusión Sanguínea. ALT y las barbas del vecino. 2006;18(3):1–40.  - Sociedad Española de Transfusión Sanguínea. Su turno. 2007;19(4):1–24.  - Sociedad Española de Transfusión Sanguínea. Relevo en la presidencia de la SETS. 2005;57(3).  - Asociación Española para el estudio del hígado. Posicionamiento de la AEEH para la eliminación de la Hepatitis C en España. Miércoles; 2018. | - | HCV Incidence and Prevalence rates were assumed to be three times higher than in the Remnant’s population for the period between 1989 and 1996. Given that donated and transfused blood started being tested since 1996, HCV Incidence values were assumed to be close to zero, as it is generally accepted that the HCV infection risk is comprised of 2/100.000 medical acts. |
| Diagnose and RIC | - Ministerio de Sanidad, Servicios Sociales e Igualdad. Informe de situación del plan estratégico para el abordaje de la hepatitis c crónica presentado al consejo interterritorial del SNS. 2017.  - Arenas C. Current Situation in Hepatitis C Drug Treatment Difficulties in Spain. FarmaJournal. 2016;1(2):165–9. | - | The annual probability of diagnosis and RIC rates were assumed to be 0% before 1994. Since that year, diagnosis values were considered until achieving an accumulated rate of 95% over the Remnant population’s HCV infection cases. A higher significance was set in the post-DAA phase, given the considerable efforts towards finding new patients. The same procedure was applied to RIC, with an accumulated value of 48% being considered for 1994 and 2014, which raised for up to 90% in subsequent years. |
| Tx1, Tx2, SVR1, SVR2 | - Mínguez C, García-Deltoro M, Flores J, Galindo M-J, Montero M, Reus S, et al. Interferon-free therapy for treating HCV in difficult-to-treat HIV-coinfected patients as implemented in routine medical practice. AIDS. 2018;1:1–34.  - Wyles D, Poordad F, Wang S, Alric L, Felizarta F, Paul Y, et al. Glecaprevir/Pibrentasvir for HCV Genotype 3 Patients with Cirrhosis and/or Prior Treatment Experience: A Partially Randomised Phase III Clinical Trial. Hepatology. 2018;67(2):514–23.  - González-Grande, R., Jiménez-Pérez, M., González Arjona, C., & Mostazo Torres, J. (2016). New approaches in the treatment of hepatitis C. World Journal of Gastroenterology, 22(4), 1421–1432. <https://doi.org/10.3748/wjg.v22.i4.1421>  - Poordad, F., Pol, S., Asatryan, A., Buti, M., Shaw, D., Hézode, C., … Mensa, F. J. (2018). Glecaprevir/Pibrentasvir in patients with hepatitis C virus genotype 1 or 4 and past direct-acting antiviral treatment failure. Hepatology, 67(4), 1253–1260. https://doi.org/10.1002/hep.29671 | - | Values for this indicator were considered to be equal to the same statistical series in Remnant’s population. |

## Appendix B - LEHC model methodology elements

Figure 2. Markov chain flowchart used in the LEHC model


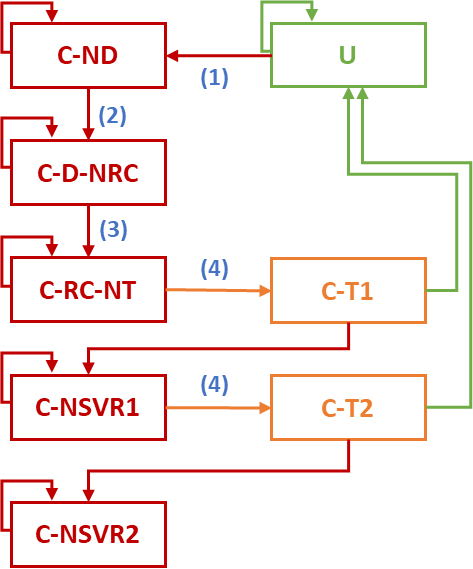


**Source:** The original figure can be consulted on the LEHC model methodological article ^14^.

**Note:** U - Uninfected (including individuals with SVR); C - chronically infected; D - diagnosed; ND - not diagnosed; NRC - not retained in care; NSVR1 - no SVR after first treatment course; NSVR2 - no SVR after second treatment course; NT - not treated; RC - retained in care; T1 - first treatment course; T2 - second treatment course; (1) - annual incidence; (2) - annual probability of diagnosis; (3) - annual probability of being retained in care; (4) - annual probability of treatment.

Figure 3. Public Health Policies considered for the LEHC model


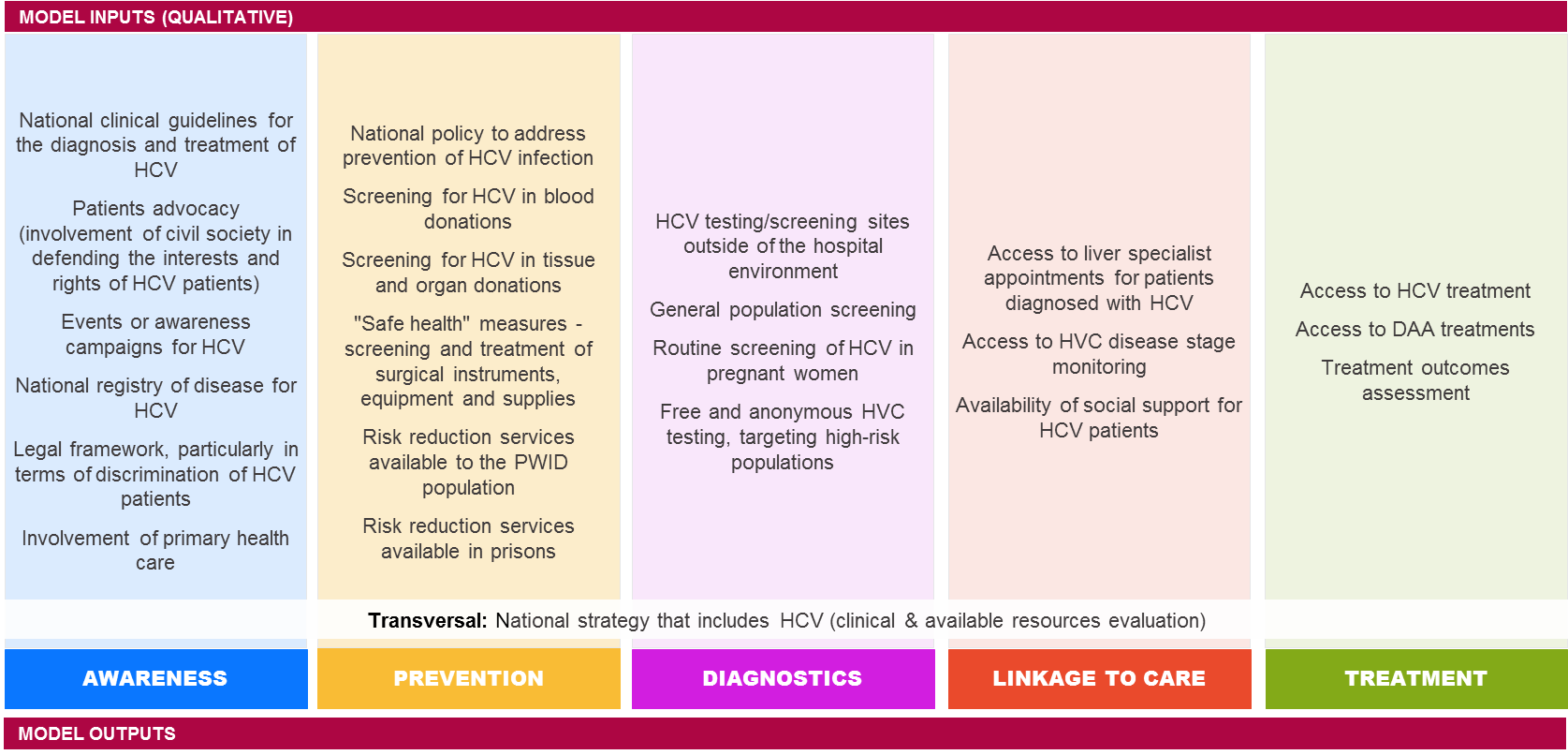


**Source:** The original figure can be consulted on the LEHC model methodological article ^14^.

Table 5. Spain’s implementation degree of the 24 Public Health Policies considered in the LEHC project

| Policy | Scale | | | | |
| --- | --- | --- | --- | --- | --- |
| *Transversal* |  | | | | |
| National strategy that includes HCV (clinical evaluation) | Not available | Available only for prevention and diagnosis | Available only for treatment | Available for prevention, diagnosis and treatment | |
| National strategy that includes HCV (available resources evaluation) | Not available | Human and financial resources attached to the strategy | Monitoring strategies available | Human, financial resources and monitoring strategies attached to the strategy available | |
| *Awareness* |  |  |  | |  |
| National clinical guidelines for the diagnosis and treatment of HCV | Not available | Available only for diagnosis | Available only for treatment | Available for diagnosis and treatment | |
| Patients advocacy (involvement of civil society in defending the interests and rights of HCV patients) | Not available | Available in a passive way | Available in an active way | - | |
| Events or awareness campaigns for HCV | Never | Rarely | Sometimes | Often | |
| National register of disease for HCV | Not available | Facultative | Mandatory | - | |
| Legal framework, particularly in terms of discrimination of HCV patients | Not available | Available only in the working environment | Available only in the framework of access to social and public services and facilities | Available in the working environment and in the framework of access to social and public services and facilities | |
| Involvement of primary health care | Not available | Available only for prevention, screening and diagnosis | Available only in patients' follow-up | Available for prevention, diagnosis, treatment and patients' follow-up | |
| *Prevention* |  |  |  | |  |
| National policy to address prevention of HCV infection | Not available / Available but not adopted | Available and partially adopted | Available and fully adopted | - | |
| Screening for HCV in blood donations | Not available | Available | - | - | |
| Screening for HCV in tissue and organ donations | Not available | Available but does not interdict donation | Available and interdicts donation | - | |
| “Safe health” measures - screening and treatment of surgical instruments, equipment and supplies | *(Cumulative scale)* | Available as methods of sterilising materials | ‘+ certification methods - physical, chemical and biological indicators | '+ validation methods | |
|  | Not available |  |  |  |  |
| Risk reduction services available to the PWID population | Not available | Needle and syringe exchange program available | Opiate Replacement Programs available | Needle and syringe exchange program and Opiate Replacement Programs available | |
| Risk reduction services available in prisons | Not available | Needle and syringe exchange program and / or safe consumption rooms available and effectively implemented | Opiate Replacement Programs available | Needle and syringe exchange program and / or safe consumption rooms effectively implemented and Opiate Replacement Programs available | |
| *Diagnostics* |  | | | | |
| HCV testing/screening sites outside of the hospital environment | Not available | Available in NGOs | Available in primary health care | Available in NGOs and primary health care | |
| General population screening | Not available | Available only in high risk ages | Universal screening | - | |
| Routine screening of HCV in pregnant women | Not available | Available | - | - | |
| Free and anonymous HVC testing, targeting high-risk populations | Not available | Available but of hard access | Available with moderate access | Widely available | |
| *Linkage to care* |  |  |  |  | |
| Access to liver specialist appointments for patients diagnosed with HCV | Not available | Available with restrictions | Available without restrictions | - | |
| Access to HCV disease stage monitoring | Not available | Available only during treatment | Available during treatment and after cure | - | |
| Availability of social support for HCV patients | Not available | Only support related to clinical questions | Only support related to non-clinical questions | Available support for clinical and non-clinical questions | |
| *Treatment* |  |  |  |  | |
| Access to HCV treatment | Available only from F4 stage | Available only from F3 stage | Available only from F1 stage | Available in all stages of disease | |
| Access to DAA treatments | Available only from F4 stage | Available only from F3 stage | Available only from F1 stage | Available in all stages of disease | |
| Treatment outcomes assessment | Not available | Available only in the pre-DAA period | Available only in the DAA period | Available, both in the pre-DAA and DAA periods | |

**Note:** The implementation degree of each policy is signalled in green highlight.

## Appendix C - Article’s Financing

Gilead financed a semiannual scholarship for a junior researcher at the University of Valencia for the data collection in Spain, three trips by the Scientific Coordination to Spain, the rental of a meeting room for the National Advisory Board, and financing to the University of Valencia that was used to purchase a computer.

This work also made use of the LEHC model, whose development and programming had in the past been financed by Gilead to the Catholic University of Portugal.

Funding from Gilead was not received by any NAB member, Scientific Coordination of the project, Local Scientific Coordination of Spain.

The article only addresses the mathematical assessment of the public health policies’ impact on hepatitis C in an open-source regime, having no connection to therapeutics, recommendation of therapeutic options, or any other commercial interest expression.
